# Welcome to open access publishing at *EvoDevo*: a macroevolutionary change in sharing data

**DOI:** 10.1186/2041-9139-1-1

**Published:** 2010-07-05

**Authors:** Mark Q Martindale, Maximilian J Telford

**Affiliations:** 1 Kewalo Marine Laboratory, University of Hawaii, Honolulu, USA; 2 Department of Genetics, Evolution and Environment, University College London, London, UK

## 

Patterns of speciation and extinction throughout the history of our planet are a testament to the complex changes in embryogenesis that have created the immense biological diversity that surrounds us.

The diversity of multicellular organisms arises through complicated and highly coordinated processes of embryogenesis that continue to be one of the most fascinating and least understood areas of biology. In contrast to other scientific disciplines, there are few theories of development that predict how complex, functional larval or adult organisms, composed of distinct and specialized cell types, arise from a single cell. The primary guiding principle in the field of developmental biology is the notion of 'differential gene expression' enunciated some 40 years ago [1] - a simple concept that explains the appearance of cells with many different phenotypes yet sharing a single genotype. Although our understanding of the control of gene expression has made huge advances, we still lack a predictive "logic" to understand the connection between genotype and phenotype. This is due, in part, to the need for consideration of the evolutionary context within which each organism has arisen.

We are in the midst of a renaissance of the rigorous study of the developmental basis of biological diversity. The field of evo-devo is gaining pace, energised by opportunities such as the freedom to go beyond the handful of genetic model systems at the cellular and molecular levels. This expansion is being fuelled by the reduced cost of nucleic acid sequencing, high throughput screening approaches, and opportunities for live cell imaging now applicable to many species of interest. Many of the recent major advances in understanding gene expression that have profound evolutionary implications (e.g. imprinting, RNAi, buffering of genetic variability) have been discovered through studies of embryonic material.

The study of development not only provides insight into the tortuous past of any one species, it illuminates the modifications in development that have given rise to existing forms. These new technologies coincide with our increasingly mature understanding of phylogenetic relationships amongst extant organisms. Interpreting developmental phenomena in this rigorous phylogenetic context is the most direct approach to gaining understanding of the cellular and molecular basis of the evolution of diversity and provides necessary information regarding the direction of evolutionary change.

All these factors have resulted in the much needed expansion of the number of new 'model' systems available for study - the new sources of data require a new and modern online venue for the communication of valuable results. The fields of developmental and evolutionary biology are inherently comparative and require the careful documentation of features of embryos that vary both across time and space and can benefit enormously from modern imaging and comparative techniques for examining increasingly large and complex data sets.

A common request from traditional print journals is for a reduction in size, colour content, or number of display figures in a manuscript or for an increased financial contribution that is unjustifiable under shrinking lab research budgets. Constraints on the ability to share high quality data are particularly limiting in these days when new model systems are being introduced to the community: often, crucial descriptive background information and detailed documentation of complex phenomena is needed to educate an eager new audience and to lay the foundation for future studies.

Clearly, web-based publications are in a position to take maximum advantage of modern technologies such as multichannel imaging, animations, time-lapse movies, and rapid access to online databases. The internet is an ideal and powerful method to access information and is available virtually everywhere in our wireless world. And that is the point: to share solid, interesting, high quality data with as many people as possible.

Open access publishing promotes rapid dissemination of information to the greatest number of individuals possible [2]. Authors of *EvoDevo *manuscripts retain copyright to their own work so they can reproduce figures and provide PDF copies of their papers on personal websites. There are no page limits or colour plate fees that artificially constrain manuscript preparation, allowing authors to provide as much information as needed to explain their results to their peers. There is a one-time article-processing charge paid at the time of publication that ensures that papers are permanently archived in multiple locations such as PubMed Central and the US National Library of Medicine, as well as in Germany, France and e-Depot [3,4]. Open access publication is now a requirement for many public funded research agencies (such as the NIH). Furthermore, online publishing at BioMed Central allows authors to rapidly track the impact of their own publications, providing metrics (number of times viewed, download statistics, etc.) for professional evaluation and advancement.

As we struggle with unscientific faith-based approaches to understanding our world, not only is evolutionary biology a cornerstone of modern biology, but also of science and intellectualism around the world. The study of development in an evolutionary and phylogenetic context, and the appreciation of the role of the developmental period in evolutionary theory, is therefore a fundamental and fascinating field of study. It is an exciting time to be involved in the field of evo-devo as the logical intersection of these basic questions with mountains of new data generated by technical advancements in the field. We invite and encourage you and your colleagues to join our outstanding group of international editors [5] in contributing to this excitement by open access publishing in *EvoDevo*.
